# Predictors of stunting among children aged 6–59 months, Zimbabwe

**DOI:** 10.1017/S1368980023000046

**Published:** 2023-04

**Authors:** Anesu Marume, Moherndran Archary, Saajida Mahomed

**Affiliations:** 1 College of Health Sciences, University of KwaZulu-Natal, Durban, South Africa; 2 Ministry of Health and Child Care, Parirenyatwa Hospital, A178 Avondale, Harare, Zimbabwe; 3 King Edward VIII Hospital, Durban, South Africa

**Keywords:** Stunting framework, Multi-sectoral, Social factors, Linear growth faltering

## Abstract

**Objective:**

Stunted children have an increased risk of diminished cognitive development, diabetes, degenerative and CVD later in life. Numerous modifiable factors decrease the risk of stunting in children. This study aimed to assess the role of the individual, household and social factors on stunting in Zimbabwean children.

**Design:**

A 1:2 unmatched case–control study.

**Setting:**

This study was conducted in two predominantly rural provinces (one with the highest national prevalence of stunting and one with the lowest prevalence) in Zimbabwe.

**Participants:**

Data were obtained from the caregivers of 150 children aged between 6 and 59 months with stunting and from the caregivers of 300 children without stunting.

**Results:**

Multiple (39) correlates of stunting were identified. Child’s age, birth length, birth weight, and weight-for-age outcome (child-related factors), caregiver’s age, maternal HIV status, occupation, and education (parental factors), breast-feeding status, number of meals, and dietary quality (dietary factors), child’s appetite, diarrhoeal and worm infection (childhood illnesses), income status, access to safe water, access to a toilet, health clubs and maternal support in infant feeding (household, socio-cultural factors) were all found to be significant predictors of childhood stunting.

**Conclusion:**

Nearly all aspects under review from the individual-, household- to social-level factors were significantly associated with childhood stunting. These findings add to the growing body of evidence supporting the WHO stunting framework and strengthen the need to focus interventions on a multi-sectoral approach to effectively address stunting in high prevalence countries.

Chronic malnutrition has a long-term impact on a child’s health with limited fatality but a diminished quality of life^(1)^. Stunting, also known as linear growth faltering, is largely irreversible. The condition can be used as a measure of the inequalities that exist within and between countries, as a result of individual, household, social, environmental and political influences^(1,2)^. Efforts to prevent stunting require a multi-sectoral approach as opposed to targeting one factor in the causality chain. There are numerous consequences of stunting including diminished cognitive development, an increased risk of CVD and degenerative diseases such as diabetes^(3)^. Childhood stunting before the age of 2 years predicts poorer cognitive and educational outcomes in later childhood and adolescence which thus has significant educational and economic consequences at the individual, household and community levels^(4)^.

Globally, stunting has declined from 32·4 % (199·5 million children) in 2000 to 21·3 % (144 million children) in 2019^(5)^. The greatest burden of stunting globally is in Africa (41 %) and Asia (53 %)^(5)^. The decrease in stunting prevalence is short of meeting the Global Nutrition Targets of 2025 and the Sustainable Development Goals of 2035^(5,6)^. Africa is the only region globally where the number of children affected with stunting has increased between 2000 and 2019^(5)^. Only 25 % of countries in Africa are on track to meet the SGD 2035 stunting targets^(5)^. Despite having a lower prevalence than some countries in Africa, one in every four children in Zimbabwe (26·5 %) is affected by stunting^(7)^. The prevalence of stunting in Zimbabwe is considerably high. Additionally, the reduction of stunting in Zimbabwe is marginal with the WHO classifying the country as off-track in meeting the Sustainable Development Goals^(5)^.

In 2013, the WHO together with UNICEF proposed a contextual framework to guide and focus on researches and interventions that aim at reducing, controlling and eliminating stunting^(2,8)^. This framework proposes a multifactorial approach towards stunting whereby stunting is described as a product of proximal and distal factors. The proximal factors include individual, maternal, household and dietary factors, diseases and infirmity. The distal factors are those factors that are largely outside an individual’s control, such as environmental, social and community factors. Multiple studies have argued that these factors are not mutually exclusive, and their cumulative contribution increases the likelihood of stunting in children^(1,9)^.

Research on the determinants of stunting utilising the UNICEF stunting framework has been conducted globally and in several African countries^(10,11)^. However, studies from Zimbabwe have considered only one or two items of the UNICEF stunting framework^(12–14)^. Therefore, the aim of this study was to assess factors across all the constructs of the UNICEF stunting framework and its association with childhood stunting in Zimbabwe.

## Methods

### Study setting

A 1:2 unmatched case–control study was conducted in Manicaland and Matabeleland South Provinces of Zimbabwe. An unmatched study was conducted due to a paucity of studies that have assessed the predictors of childhood stunting in Zimbabwe. An unmatched study is less costly due to reduction in time required in identifying study participants. Additionally, unmatching reduces bias^(15)^. The provinces were purposively selected for having the highest (Manicaland) and lowest (Matabeleland South) prevalence of childhood stunting. Districts with the highest stunting prevalence in Manicaland and with the lowest prevalence in Matabeleland South were selected.

Manicaland often referred to as Eastern Highlands/Eastern Zimbabwe is a mountainous region characterised by very high relief rainfalls. The province is the second most populated province in Zimbabwe with 1 752 698 people. The main source of income in Manicaland Province is through mining (gold and diamond), plantations (tree, tea, banana, macadamia and coffee), commercial and subsistence farming. Individual households in the province rely mainly on crop production and employment within the plantations and mining (commercial and artisanal). The province is the most affected by nearly all forms of childhood malnutrition with the highest prevalence of stunting (31·2 %), acute malnutrition (mid-upper arm circumference (MUAC) < 125 mm) (0·53 %), severe acute malnutrition (MUAC < 115 mm) (0·5 %) and overweight (4·5 %) in the country. Four of the seven districts in Manicaland Province are among the top ten districts with the highest prevalence of stunted children in Zimbabwe^(7)^.

Matabeleland South Province has a population of 824 463 people and is the least populated province in Zimbabwe located in the south-western part of the country. The main source of income in this province is cross-border trading, livestock and subsistence farming. The province is characterised by frequent droughts as it is at the edge of the Kalahari Desert. The province is the least affected by nearly all forms of childhood malnutrition in Zimbabwe according to the 2018 National Nutrition Survey characterised by the least prevalence of overweight (1·7 %) and stunting (24·2 %). Three of the seven districts in Matabeleland South province are among the ten districts with the lowest prevalence of stunting in Zimbabwe^(7)^. Participants were drawn from the four districts in Manicaland with the highest prevalence of stunting and from three districts in Matabeleland with the lowest prevalence of stunting. The selection of these districts ensured representation of children with stunting from the areas that are most and least affected.

### Study population and sampling

The sample size was estimated using the chi-squared test to be at least 450 and to detect a smaller effect size of at least 0·185 with 90 % power based on the G Power 3.1.9.7 sample size calculation software. Sample size calculation assumed a 95 % level of precision, at 95 % confidence with the assumption that the population thus identified has the least variability in the attributes being measured. Assigning one case for two controls, the sample required was 150 cases and 300 controls. The 1 case: 2 controls ratio was used to increase the statistical power and to try controlling for unmatched confounders. A case was defined as a child aged 6–59 months whose height-for-age was below –2 sd on the WHO Child Growth standards at the time of data collection. Inclusion in the study required that the caregiver or mother was able to respond to all aspects under consideration. Children were excluded if they were known to have a condition associated with growth inhibition such as disproportionate dwarfism (identified through distinct facial and skeletal features such as a disproportionately large head). A control was defined as a child aged 6–59 months who lived within the same administrative ward as a case and whose height-for-age was above the –2 sd line at all readings since birth. Both cases and controls were recruited on the basis that they were residents in the selected district for at least 3 months.

A register maintained by the village health workers (VHW) of all children within their catchment area that contained anthropometric measurements and immunisation data was reviewed. Children who had a Z-score of –2 sd on the WHO child growth curve at the time of data collection were identified and populated into a sampling frame. The number of cases per district was identified by dividing the total number expected with the number of districts under consideration in each province. The number found was regarded as the skip pattern (*k*). Every *k* ^th^ child was recruited as a case in our study. A child who resided within ten households from an identified case who satisfied the inclusion criteria for a control was enrolled. Call-backs were done in instances where the child and/or caregiver were not available during data collection. The child was replaced if it was not possible to obtain data during the time that the data collection team was operating within the district.

### Data collection tools

A questionnaire was designed in the indigenous languages of the two communities (Shona for Manicaland and Ndebele for Matabeleland South) from an English template. The English template was developed using the stunting conceptual framework and based on previous studies that assessed predictors of childhood stunting^(16,17)^. Each of the constructs of the stunting framework had a set questions^(2)^. The constructs considered were maternal factors, household factors, child feeding, breast-feeding, child illnesses, water, sanitation and social factors. A checklist was used to collect additional data from the ante-natal care (ANC) records of the mother and the child’s health card. From the ANC records, maternal data such as the mother’s age, HIV status, gestational age at first ANC booking and place of delivery were collected. Data from the child’s health card included the child’s immunisation status, date of birth, HIV status, weight-for-age, MUAC and height-for-age.

### Variables

Exposure variables were derived from the UNICEF stunting framework^(2)^. The categories household and family factors, complementary feeding, breast-feeding, infection and community and societal factors were considered in the analysis. Subsections, agriculture and food systems, political economy and education, were not considered in the current study as they are best conducted in a secondary data analysis. The variables weight-for-age and wasting while being in the framework were used to measure the presence of co-morbidity between stunting and other forms of malnutrition. Before inclusion of these variables in the regression model, it was noted that there was no collinearity between stunting and any malnutrition variables in the present study.

The DHS wealth index guided calculation of the household wealth index^(18)^. The wealth index was calculated using principal components analysis to generate five quintiles which were collapsed to three to avoid categories with less than five respondents. The quintiles were calculated using household asset data. Variables included when calculating the wealth index were ownership of assets (movable and immovable), type of dwelling structure, type of roofing and flooring, water and sanitation facilities, size of agricultural land and quantity of livestock. The study was carried out predominantly in the rural area; thus, a set of items common to rural households was developed. Factor scores were then developed for the households with consideration of earlier highlighted indicators. The factor scores were used to develop a 3-factor wealth index that takes into consideration differences between provinces and areas through a regression on the common factor scores. The household wealth index was calculated using SPSS. Development of household wealth index followed the steps in the DHS guide for developing household wealth indices^(18)^. Wealth indices have been noted to be a more reliable measure of chronic malnutrition in comparison to either monthly consumption or income due to both indicators (wealth indices and chronic malnutrition) being long-term^(19)^.

Water collected from boreholes, tap and protected wells was qualified as safe water sources while that collected from shallow wells, rivers and streams was regarded as unprotected water. Vaccination was measured checking the child health card to assess if a child’s vaccination status was up to date. The immunisation schedule in Zimbabwe starts with neonatal vaccination with the Bacillus Calmette-Guerin vaccine for protection against TB, followed by a set of four vaccines (rotavirus vaccine, pneumonia conjugate vaccine, pentavalent vaccine, oral polio vaccine) at 6, 10 and 14 weeks and lastly the measles and rubella vaccine at 9 months, with option of booster shots of the diphtheria, tetanus, pertussis vaccine and oral polio vaccine at 18 months. The pentavalent vaccine protects against diphtheria, tetanus, pertussis, hepatitis B and Haemophilus influenza type B.

The child’s diet was assessed by asking caregivers to describe all food items that their child consumed in the last 7 d using a FFQ adopted and adapted from the recommended Food and Administration Organization FFQ. Children were then classified as having a four-star diet as that is the commonly used term in health education for mothers and communities in Zimbabwe due to it being simple and easy to remember^(20,21)^. The four-star diet was defined as a day’s consumption of meal or meals that contain all the four food groups: animal-source foods (flesh, eggs, milk and milk products), staples (grains, roots and tubers), legumes and vitamin rich foods (fruits and vegetables).

There are multiple clubs that operate in communities with several functions that directly or indirectly influence child health. Health facility-based clubs are formed by VHW mainly to provide health education on pregnancy and child health issues to mothers or caregivers in the catchment area. In addition, there were also church-based clubs, social and financial clubs. Some caregivers could be a member of more than one club. Caregivers were asked if they were a member of any club.

### Data collection process

Data were collected between June and August 2020. Final year BSc Nutrition students at the University of Zimbabwe were recruited and trained for the data collection as research assistants. The research assistant used the VHW register in selection of both cases (150) and controls (300). An android-based questionnaire was used to interview mothers/caregivers of children under-five years of age within Manicaland and Matabeleland South Provinces. The child’s weight, MUAC and height were measured using calibrated tools (measuring scale, MUAC tape and height board, respectively). The WHO child growth standards were used to determine the nutrition status of children^(22)^. The principal investigator conducted daily quality checks while the research assistants collected data to ensure that the correct procedures were being followed and to assess the quality of data being collected. On-job training was done where research assistants were having challenges following the data collection guidelines and processes.

### Anthropometric measurements

Standing height was measured for children older than 24 months. Length was measured for children 6–23 months old and those who were not able to stand. Children were positioned at the Frankfurt plane using a wooden height-board graduated and calibrated to read to the nearest 0·1 cm. While identifying cases and controls from the VHW register, any child misclassified as stunted was replaced using the skip pattern described. Children’s weight was measured using a calibrated measuring scale. Calibration of the weight scale was done using calibration weights while that of the height board was calibrated using a calibration rod of known and fixed length.

### Data analysis

Data were populated into an excel spreadsheet by the mobile phone application. The excel spreadsheet was exported into R statistical package for data analysis. Mean age of children was calculated while the median was used in describing the age of the mother and father. Frequencies were used to describe socio-demographic data. Children’s height-for-age and weight-for-age outcomes were compared with the WHO growth charts in use in Zimbabwe. Children with a Z-score value below the –2 sd Z-score line of the WHO growth standards were categorised as stunted (height-for-age) or underweight (weight-for-age). To control for confounding, multivariate logistic regression was conducted on all variables identified. Variables identified through multivariate logistic analysis with a *P*-value ≤ 0·05 were regarded as statistically significant predictors of stunting. Stepwise backward logistic regression was conducted to identify variables which were strongly associated with stunting.

## Results

A total of 450 children were enrolled in the study. The mean age for all the children was 17·6 months (sd ± 12·7). Cases (mean 14·2 months, sd ± 10·5) were significantly younger than controls (mean 19·3 months, sd ± 13·3) (*P* < 0·001). A larger proportion of the children were boys (54·4 %). The majority of respondents (92 %) resided in rural areas. More than 18 % of the respondents had no toilet facilities in their household with a majority (83 %) of those stating that they utilised bush systems. Just over half the sample accessed their water from a borehole (55 %) while only 5·5 % had access to tap water. A high proportion (59·8 %) of the respondents reported having one form of farming land (communal or commercial) from which they provided food for their family. More than half of the mothers (51 %) interviewed stated that they had weaned or were planning to wean their children on or after 24 months of age (Table 1).

**Table 1:** Socio-demographic characteristics of the children and caregivers

	Cases (*n* 150)		Controls (*n* 300)		Total (*n* 450)		*P*-value
	*n*	%	*n*	%	*n*	%	
Province							1·000
Manicaland	75	50·0	150	50·0	225	50·0	
Matabeleland south	75	50·0	150	50·0	225	50·0	
Child factors							
Child sex							0·462
Female	72	48·0	133	44·3	205	45·6	
Male	78	52·0	167	55·7	245	54·4	
Child’s age (months)							< 0·001
Mean	14·2		19·3		17·6		
sd	10·5		13·3		12·7		
Weight-for-age							
Underweight (< 2 sd Z-scores)	21	14·0	102	34·0	123	27·3	< 0·001
Overweight (> 2 sd Z-scores)	38	25·3	51	17·0	89	19·8	0·036
Wasting							< 0·001
Normal	126	84·0	284	94·7	410	91·1	< 0·001
Wasted (< 125 mm MUAC)	24	16·0	16	5·3	40	8·9	< 0·001
Birth weight							< 0·001
Normal	99	66·0	259	86·3	358	79·6	< 0·001
Low-birth-weight (< 2·5 kg)	51	34·0	41	13·7	92	20·4	< 0·001
Median	3·16		2·80		3·00		< 0·001
Q1–Q3	2·90–3·50		2·50–3·00		2·65–3·40		
Birth length							< 0·001
Normal	118	78·7	275	91·7	393	87·3	< 0·001
Short-for-gestation-age (< 46 cm)	32	21·3	25	8·3	57	12·7	< 0·001
Median	50·0		49·0		50·0		< 0·001
Q1–Q3	49·0–52·0		46·0–50·0		48·0–51·0		
Child HIV status							0·762
Negative	143	95·3	284	94·7	427	94·9	
Positive	7	4·7	16	5·3	23	5·1	
Maternal factors							
Mother’s age (years)							< 0·001
Median	26·0		25·0		25·0		
Q1–Q3	22·0–31·8		21·0–31·0		21·0–31·0		
Maternal HIV status							0·576
Negative	114	76·0	235	78·3	349	77·6	
Positive	36	24·0	65	21·7	101	22·4	
Mother education							< 0·001
No school	7	4·7	9	3·0	16	3·6	0·070
Primary	51	34·0	95	31·7	146	32·4	< 0·001
Secondary	88	58·7	188	62·7	276	61·3	< 0·001
Tertiary	4	2·7	8	2·7	12	2·7	0·002
Maternal occupation							< 0·001
Formal	6	0·7	56	18·7	57	12·7	< 0·001
Informal	68	48·0	58	19·3	130	28·9	< 0·001
Unemployed	76	51·3	186	62·0	263	58·4	0·033
Paternal factors							
Father’s age (years)							< 0·001
Median	32·0		31·0		31·0		
Q1–Q3	26·0–38·0		26·0–39·0		26·0–38·0		
Father occupation							< 0·001
Formal	36	24·0	128	42·7	164	36·4	< 0·001
Informal	45	30·0	75	25·0	120	26·7	0·261
Unemployed	69	46·0	97	32·3	166	36·9	0·010
Father education							< 0·001
No school	11	6·7	8	2·7	18	4·0	0·070
Primary	49	36·0	40	13·3	94	20·9	< 0·001
Secondary	82	56·0	225	75·0	309	68·7	< 0·001
Tertiary	8	1·3	27	9·0	29	6·4	0·002
Household factors							
Number of siblings							0·026
Only child	32	21·3	94	31·3	126	28·0	
Multiple	118	78·7	206	68·7	324	72·0	
Number in household							0·010
Less than 4 people	59	39·3	82	27·3	141	31·3	0·026
More than 4 people	91	60·7	218	72·7	309	68·7	0·026
Median	5·00		5·00		5·00		0·132
Q1–Q3	4·00–7·00		4·00–7·00		4·00–7·00		
Child spacing							< 0·001
Less than 24 months	24	20·3	130	63·1	154	47·5	< 0·001
More than 24 months	94	79·7	76	36·9	170	52·5	< 0·001
Parental survival							0·052
Both parents alive	135	90·0	241	80·3	376	83·6	
Both parents deceased	10	6·7	36	12·0	46	10·2	
Father only alive	2	1·3	5	1·7	7	1·6	
Mother only alive	3	2·0	18	6·0	21	4·7	
Social and cultural factors							
Religion							0·658
Christianity	126	84·0	247	82·3	373	82·9	
Other	24	16·0	53	17·7	77	17·1	
Mother’s club							< 0·001
Member	87	58·0	123	41·0			
Non Member	63	42·0	177	59·0			
Water and sanitation factors							
Toilet							< 0·001
Blair toilet	83	55·3	217	72·3	300	66·7	
Flush toilet (water closet)	25	16·7	52	17·3	77	17·1	
No toilet	42	28·0	31	10·3	73	16·2	
Water source							0·005
Protected	89	59·3	217	72·3	306	68·0	0·014
Not protected	61	40·7	83	27·7	144	32·0	0·014
Distance to water source							0·408
Near (≤ 5 km)	106	70·7	223	74·3	329	73·1	
Far (> 5 km)	44	29·3	77	25·7	121	26·9	
Rubbish pit	121	80·7	286	95·3	407	90·4	< 0·001
Economic and livelihood factors							
Floor material							< 0·001
Cement	82	54·7	231	77·0	313	69·6	< 0·001
Cow dung	34	22·7	32	10·7	66	14·7	0·002
Earth	14	9·3	23	7·7	37	8·2	0·466
Tile	20	8·0	14	4·7	34	5·1	0·128
Fuel for cooking							0·549
Electricity	11	7·3	27	9·0	38	8·4	
Firewood	139	92·7	273	91·0	412	91·6	
Mobile phone ownership	141	94·0	273	91·0	414	92·0	0·269
Farming land							0·038
No farming land	70	46·7	111	37·0	81	18·0	
Communal	68	45·3	165	55·0	233	51·8	
Commercial	12	8·0	24	8·0	36	8·0	
Wealth index							< 0·001
High	52	34·7	38	12·7	90	20	
Middle	42	28·0	228	76·0	270	60	
Low	56	37·3	34	11·3	90	20	

MUAC, mid-upper arm circumference.

### Household and family factors

Low-birth-weight children and children born short for their gestation age were 4 times (*P* = 0·015) and 1·4 times (*P* = 0·033) more likely to become stunted as they grow (Table 2). There was a significant 1·4 times likelihood that children who were underweight children were also stunted; however, there was no significant association between being overweight and stunting (*P* = 0·266). Middle-aged mothers and fathers (25–35 years) had higher odds of having children affected with stunting (*P* = 0·053 and *P* = 0·022, respectively). Children of HIV-infected mothers were 3·6 times more likely to be stunted (*P* = 0·027). Children of mothers who reported not having gone to school were significantly more likely to be stunted (*P* = 0·012) and similarly father’s education status had a 1·7 increase in the likelihood of a child being stunted (*P* = 0·008). Maternal employment status was not significantly associated with childhood stunting. However, paternal unemployment put children at a 2·3 times increased risk of stunting (*P* = 0·008). Children whose parents had both deceased had increased odds of being stunted (adjusted OR 2·20 95 % CI 1·03, 4·78). Having more than two siblings was a significant risk factor for stunting (*P* = 0·030). Children born less than 24 months apart were 8 times more likely to be stunted in comparison to being an only child (*P* < 0·001) (Table 2).

**Table 2:** Predictors of early childhood (6–59 months) stunting in Zimbabwe, logistic regression analysis

Variable	Cases (*n* 150)		Control (*n* 300)		Crude			Adjusted		
	*n*	%	*n*	%	OR	95 % CI	*P*-value	OR	95 % CI	*P*-value
Household and family										
Child-related factors										
Province										
Matabeleland South	75	50·0	150	50·0	0·98	0·66, 1·46	0·920	0·74	0·38, 1·43	0·371
Manicaland	75	50·0	150	50·0	1			1		
Child’s age										
Mean age					0·96	0·94, 0·98	< 0·001	0·95	0·92, 0·97	< 0·001
Sex										
Male	78	52·0	167	55·7	0·86	0·56, 1·24	0·364	0·57	0·30, 1·07	0·082
Female	72	48·0	133	44·3	1			1		
Birth weight										
Low birth weight	51	34·0	41	13·7	3·25	2·03, 5·22	< 0·001	4·00	1·96, 21·62	0·015
Birth weight > 2·5 kg	99	66·0	259	86·3	1			1		
Birth length										
Short for gestation age	32	21·3	25	8·3	2·98	1·69, 5·25	< 0·001	1·39	1·16, 3·92	0·033
Birth length > 46 cm	118	78·7	275	91·7	1			1		
Wasting										
MUAC < 125 cm	24	16·0	16	5·3	3·38	1·78, 6·81	< 0·001	2·37	0·72, 8·02	0·157
MUAC ≥ 125 cm	126	84·0	284	94·7	1			1		
Weight-for-age										
Underweight	21	14·0	102	34·0	1·89	0·94, 3·80	0·070	1·42	1·28, 6·52	< 0·001
Overweight	38	25·3	51	17·0	6·85	3·52, 13·32	< 0·001	1·53	0·72, 3·28	0·266
Normal weight	16	10·7	147	49·0	1			1		
Maternal factors										
Maternal age (years)										
< 18	6	2·0	26	8·7	0·12	0·02, 0·85	0·019	0·91	0·87, 0·96	< 0·001
18–24	80	61·3	72	24·0	1·28	0·25, 6·52	0·768	0·73	0·18, 2·96	0·655
25–35	46	30·7	151	51·7	0·30	0·06, 1·52	0·124	1·62	0·99, 2·65	0·053
36–45	7	4·0	43	14·7	0·14	0·02, 0·84	0·017	0·42	0·22, 0·80	0·007
> 45	11	2·0	5	1·0	1					
Maternal HIV status										
Positive	36	24·0	65	21·7	1·14	0·72, 1·82	0·576	3·63	1·15, 11·46	0·027
Negative	114	76·0	235	78·3	1			1		
Maternal marital status										
Cohabiting	21	14·0	39	13	1·04	0·55, 1·82	0·957	0·97	0·52, 1·80	0·934
Single	19	12·7	53	17·7	0·69	0·39, 1·29	0·286	0·75	0·40, 1·35	0·348
Divorced	12	8·0	19	6·3	1·22	0·57, 2·61	0·612	1·66	0·37, 12·21	0·178
Married	98	65·3	189	63	1			1		
Maternal occupation										
Formal	6	0·7	56	18·7	1			1		
Informal	68	48·0	58	19·3	69·52	9·34, 517·50	< 0·001	0·35	0·16, 0·73	0·005
Unemployed	76	51·3	186	62·0	23·18	3·15, 170·48	< 0·001	0·60	0·32, 1·14	0·119
Maternal education										
No school	7	4·7	9	3·0	16·88	3·05, 93·39	< 0·001	1·73	1·02, 5·72	0·012
Primary	51	34·0	95	31·7	18·23	4·09, 81·14	< 0·001	0·88	0·28, 2·89	0·822
High school	88	58·7	188	62·7	5·04	1·17, 21·66	0·017	0·57	0·19, 1·83	0·325
Tertiary	4	2·7	8	2·7	1			1		
Paternal factors										
Paternal age										
< 18	8	2·0	6	1·3	25·25	2·43, 35·44	< 0·001	1·01	0·98, 1·03	0·526
18–24	57	44·0	5	0·3	2·57	1·56, 4·23	< 0·001	0·06	0·01, 0·53	0·011
25–35	71	51·3	142	49·3	20·29	2·74, 30·53	< 0·001	4·67	1·59, 13·70	0·022
36–45	5	2·0	108	36·0	1·08	0·11, 10·73	0·945	0·55	0·32, 12·50	0·511
> 45	9	0·7	39	13·0	1			1		
Paternal occupation										
Formal	36	24·0	128	42·7	1			1		
Informal	45	30·0	75	25·0	2·13	1·26, 3·6	0·004	1·03	0·62, 1·70	0·921
Unemployed	69	46·0	97	32·3	2·53	1·56, 4·09	< 0·001	2·31	1·01, 4·66	0·008
Paternal education										
No school	11	6·7	8	2·7	16·88	3·05, 93·39	< 0·001	6·15	1·95, 9·68	0·016
Primary	49	36·0	40	13·3	18·23	4·09, 81·14	< 0·001	3·31	0·82, 22·37	0·136
Secondary	82	56·0	225	75·0	5·04	1·67, 16·76	0·227	2·55	0·65, 16·86	0·235
Tertiary	8	1·3	27	9·0	1			1		
Household factors										
Parental survival										
Both parents alive	123	90·0	240	80·3	1			1		
Father only alive	5	1·3	6	1·7	0·71	0·02, 3·05	0·471	1·66	0·03, 61·45	0·794
Mother only alive	9	2·0	18	6·0	0·30	0·07, 0·90	0·055	0·86	0·16, 3·50	0·849
Both deceased	13	6·7	36	12·0	0·50	0·67, 2·55	0·402	2·20	1·03, 4·78	0·043
Number in household										
< 3 people	28	18·7	37	12·3	1			1		
3–4 people	31	20·6	45	15·0	0·91	0·47, 1·78	0·784	0·72	0·14, 2·68	0·225
> 4 people	91	60·7	218	72·7	0·55	0·38, 0·88	0·011	1·56	0·74, 3·40	0·253
Siblings age difference										
< 24 months	94	62·7	76	25·3	3·63	2·20, 6·00	< 0·001	8·00	4·15, 16·24	< 0·001
≥ 24 months	24	16·0	130	43·3	0·54	0·30, 0·98	0·041	0·83	0·88, 1·32	0·091
Only child	32	21·3	94	31·3	1			1		
Number of siblings										
Only child	32	21·3	94	31·3	1			1		
1–2 siblings	11	7·3	88	29·3	2·72	1·29, 5·73	< 0·001	2·56	1·93, 6·59	0·005
> 2 siblings	101	67·3	118	39·3	0·39	0·23, 0·63	0·009	6·15	2·95, 19·68	0·030
Child feeding										
Breastfed										
Yes	137	91·3	293	97·7	1			1		
Never	13	8·7	7	2·3	3·97	2·26, 5·19	< 0·001	2·88	1·89, 3·42	< 0·001
Breast-feeding initiation										
Within 1 h	61	40·7	155	51·7	1			1		
After 1 h	89	59·3	145	48·3	1·56	1·05, 2·32	0·028	1·17	0·85, 1·64	0·331
Age starting complementary feed										
< 6 months	83	55·3	160	53·3	1·08	0·73, 2·29	0·638	1·94	1·71, 5·64	0·018
≥ 6 months	67	44·7	140	46·7	1			1		
Meals per day										
< 3 meals	89	59·3	195	65·0	1·05	0·51, 1·17	0·215	1·52	1·13, 12·87	< 0·001
≥ 3 meals	61	40·7	105	35·0	1			1		
Four-star diet										
All components	54	36·0	168	56·0	1			1		
Missed one or more foods	96	64·0	132	44·0	2·26	1·32, 3·74	< 0·001	1·38	1·23, 5·63	< 0·001
Infection										
Child HIV status										
Positive	7	4·7	16	5·3	0·87	0·35, 2·28	0·895	0·59	0·11, 2·94	0534
Negative	143	95·3	284	94·7	1			1		
Appetite loss last month										
Never	58	38·7	136	45·3	1			1		
Rarely	68	45·3	132	44·0	1·21	0·78, 1·86	0·395	1·12	0·69, 1·81	0·642
Frequent	24	16·0	32	10·67	1·76	0·96, 3·29	0·066	1·78	1·03, 3·40	0·019
Child illness past month										
No illness	32	21·3	89	29·7	1			1		
Diarrhoea last month	104	69·3	92	30·7	3·14	2·49, 6·06	< 0·001	5·91	2·89, 12·65	< 0·001
Worm infection	13	8·7	6	2·0	6·03	1·82, 13·66	0·002	4·79	1·06, 23·48	0·046
Respiratory infection	63	42·0	63	21·0	2·78	1·63, 4·89	0·004	1·03	0·83, 1·29	0761
Fever	65	43·3	96	32·0	1·88	1·09, 2·43	0·018	1·27	0·52, 3·08	0·603
Health and healthcare										
Place of delivery										
Health facility	111	74·0	258	86·0	1			1		
Home	39	26·0	42	14·0	2·16	1·32, 3·52	0·003	3·39	2·16, 10·94	0·037
Distance health facility										
< 5 km	89	59·3	156	52·0	1			1		
≥ 5 km	61	40·7	144	48·0	0·71	0·47, 1·06	0·094	0·75	0·38, 1·47	0·409
ANC attendance and visits										
Did not attend	13	8·7	9	3·0	1·5	60·64, 3·83	0·329	2·86	1·59, 9·91	0·011
< 4 visits	50	33·3	202	67·3	0·27	0·22, 0·49	< 0·001	4·03	1·11, 11·24	< 0·001
≥ 4 visits	87	58·0	94	31·3	1			1		
Vitamin A doses										
Never received vitamin	34	22·7	43	14·3	2·30	1·33, 3·92	0·002	0·86	0·22, 3·27	0·820
Missed vitamin A doses	52	34·7	71	23·7	2·13	1·10, 4·64	< 0·001	0·74	0·24, 2·23	0·590
Received adequate doses	64	42·7	186	62·0	1			1		
Vaccination										
Never been vaccinated	18	12·0	11	3·7	3·63	1·65, 8·61	< 0·001	3·22	1·04, 8·78	0·023
Missed vaccine antigens	41	27·3	87	29·0	1·05	0·59, 2·40	0·658	1·19	0·95, 1·45	0·133
Vaccines up-to-date	91	60·7	202	67·3	1			1		
Household wealth										
Floor material										
Cow dung	34	22·7	32	10·7	0·74	0·32, 1·72	0·487	1·42	1·03, 2·33	0·042
Earth	14	9·3	23	7·7	0·43	0·16, 1·10	0·077	1·25	0·89, 2·96	0·306
Cement	82	54·7	231	77·0	0·25	0·12, 0·51	< 0·001	0·60	0·40, 0·90	0·013
Tile	20	13·3	14	4·7	1			1		
Farming land										
No farming land	70	46·7	111	37·0	1·26	0·59, 2·68	0·546	1·24	0·61, 2·67	0·569
Communal	68	45·3	165	55·0	0·82	0·39, 1·74	0·612	0·37	0·18, 0·72	0·004
Commercial	12	8·0	24	8·0	1			1		
Wealth index										
High	52	34·7	38	12·7	1			1		
Average	42	28·0	228	76·0	1·79	0·57, 3·49	0·148	6·81	0·23, 8·97	0·365
Low	56	37·3	34	11·3	2·31	1·69, 3·25	0·051	3·34	1·38, 7·43	< 0·001
water and sanitation										
Water source										
Protected	89	59·3	217	72·3	1			1		
Unprotected	61	40·7	83	27·7	1·79	1·19, 2·71	0·005	1·42	1·29, 5·89	0·029
Distance to water source										
Far	44	29·3	77	25·7	1·20	0·78, 1·86	0·408	0·95	0·77, 1·18	0·671
Near	106	70·7	223	74·3	1			1		
Type of toilet										
No toilet	42	28·0	31	10·3	2·82	1·45, 5·48	0·002	0·80	0·21, 3·08	0·479
Blair toilet	83	55·3	217	72·3	0·86	0·46, 1·37	0·406	0·30	0·18, 0·50	< 0·001
Water closet	25	16·7	52	17·3	1			1		
Rubbish pit										
Available	121	80·7	286	95·3	1			1		
No rubbish pit nearby	29	19·3	14	4·7	4·90	2·50, 9·60	< 0·001	2·15	1·16, 6·39	< 0·001
Society and culture										
Religion										
Christianity	126	84·0	247	82·3	1			1		
Any other	24	16·0	53	17·7	0·89	0·50, 1·48	0·622	0·81	0·32, 2·02	0·661
Health seeking behaviour										
No delay	97	64·7	239	79·7	1			1		
First seek alternative care	19	12·7	38	12·7	0·81	0·45, 1·48	0·494	0·62	0·21, 1·95	0·398
Do not go to health facility	11	7·3	25	8·3	0·92	0·44, 1·95	0·832	0·76	0·14, 3·85	0·742
Cultural influences on diet										
No influence	63	42·0	233	77·7	1			1		
Pre-lacteal feeding	72	48·0	49	16·3	5·43	2·24, 6·04	< 0·001	7·06	3·33, 14·96	< 0·001
Meat, eggs not suitable	15	10·0	28	9·3	1·98	1·21, 7·29	< 0·001	2·05	0·90, 4·67	0·087
Mother’s club										
Not in any club	90	63·3	178	56·3	0·72	0·49, 1·03	0·072	1·48	0·38, 3·27	0·139
Church-based clubs	21	14·0	37	12·3	0·64	0·35, 1·16	0·138	0·62	0·21, 1·95	0·398
Agriculture-based clubs	11	7·3	31	10·3	1·02	0·49, 2·13	0·953	0·76	0·14, 0·85	0·013
VHW led clubs	78	52·0	215	71·7	1			1		
Support with child care										
Maternal grandmother	82	54·7	190	63·3	1·12	0·79, 1·58	0·539	0·38	0·19, 0·63	0·023
Aunt	86	57·3	150	50·0	0·84	0·59, 1·19	0·333	0·98	0·86, 1·11	0·706
Child’s siblings	80	54·3	160	53·3	0·96	0·67, 1·37	0·835	0·57	0·19, 1·83	0·325
Paternal grandmother	68	45·3	149	49·7	1·06	0·73, 1·52	0·776	0·61	0·11, 3·16	0·558
Church member	5	3·3	9	3·0	0·87	0·28, 2·65	0·802	0·69	0·23, 2·20	0·511
Spouse	104	69·3	216	72·0	1			1		

ANC, ante-natal care; VHW, village health worker.

### Breast-feeding and complementary feeding

A majority of the children (63·8 %) were still breast-feeding at the time of the interview. Children who had never been breastfed were 3 times more likely to be stunted in comparison to children with any history of breast-feeding (*P* < 0·001). Children who had consumed a diet which lacked animal source foods within the previous 7 d were significantly more likely to be stunted in comparison to those who had consumed a diet with all the components of the four-star diet (*P* < 0·001). Children who consumed less than 3 meals/d in the past 7 d were 1·5 times more likely to have stunted growth (*P* < 0·001). Early introduction of complementary feed (before 6 months) increased the risk of stunted growth in children 1·9 times (*P* = 0·018) (Table 2).

### Infection

Children with reported diarrhoeal infection in the past 1 month were 5·9 times more likely to be identified with stunting in comparison with no reported infection in the same period (*P* < 0·001). Reported worm infection in the past month increased the odds of children being identified as having stunted growth (*P* = 0·046). Children reported as having frequent loss of appetite within the past month were 1·8 times more likely to be affected with stunting (*P* = 0·019) (Table 2).

### Health and healthcare

Children delivered at home were 3 times more likely to be stunted in comparison with children delivered at the health facility (*P* = 0·037). Children of mothers who did not go to the health facility for ANC services while pregnant and of mothers who accessed ANC for less than 4 visits had increased odds of being stunted (adjusted OR 2·86 95 % CI 1·59, 9·91 and adjusted OR 4·03 95 % CI 1·11, 11·24, respectively). Children with no history of vaccination were 3·2 timely more likely to be stunted (*P* = 0·023) (Table 2).

### Poverty, income and wealth

In comparison with a house with floor tiles, children who lived in a house with floors made using cow dung had a 1·42 likelihood of being stunted (*P* = 0·042). Having no farm land increased a child’s odds of being stunted 1·4 times (*P* = 0·042). Children from low families identified within the low wealth index were 3·34 times more likely to be identified as stunted (*P* < 0·001) (Table 2).

### Water and sanitation

Drinking water from unprotected water sources was a significant risk factor for childhood stunting (*P* = 0·029). More than a quarter (27 %) of the respondents reported that they had to walk more than 1 km to access safe water (Table 2). Living in a house without a functional toilet and at a household far from water sources had no significant relationship with a child’s stunting diagnosis. A household without a rubbish pit increased the likelihood of childhood stunting 2·2 times (*P* < 0·001).

### Society and cultural factors

A proportion of the respondents (13 %) stated that they would seek alternative care (religious and traditional health systems) before going to the health facility. Early booking of pregnancy was the most affected by cultural practices with more than a third of the respondents (34 %) stating that a woman should go for ANC only when the pregnancy is showing. Children who were given pre-lacteal feed were 7 times more likely to be stunted (*P* < 0·001). Children whose culture discouraged consumption of meat and eggs were 2 times more likely to be stunted (*P* = 0·087). The most common support systems for child care were the maternal grandmother, aunt, siblings, paternal grandmother and church member. Support from the maternal grandmother was significantly protective of stunting (*P* < 0·023). More than half of the caregivers interviewed (58 %) were a member of one form of mother’s club. The most common mother’s clubs were those initiated by VHW (62 %), followed by church clubs (13 %) and projects and financial clubs (9 %). Being a member of the club that focused on projects and financial assistance was protective from childhood stunting (*P* = 0·013).

## Discussion

Stunting is a complex nutrition outcome with a multifaceted causal chain. This study assessed all the categories described on the UNICEF stunting framework with exception of three subcategories: political economy, education and agriculture and food systems. The study is the first in Zimbabwe to utilise the framework. All categories were identified as having significant predictors of childhood stunting among the Zimbabwean children.

Multiple studies and nationwide surveys have found boys to have a higher likelihood of being stunted than girls^(23,24)^. However, we found no association between a child’s sex and stunting which may be due to a smaller sample size in comparison with many of the other studies. Our study found the mean age of cases significantly lower in comparison with controls. Linear growth retardation occurs mainly in the first 1000 d and thus increasing the likelihood that the children identified as cases would be younger^(25)^. However, while linear growth faltering may start at an early age, it is more apparent as children grow older. Low-birth-weight children and those born short-for-gestation-age had increased odds for malnutrition. A study conducted in Canada found a relationship between children born short-for-gestation-age and low levels of the insulin-like growth factor I^(26)^. The finding confirms the presence of both genetic and epigenetic influences towards childhood stunting.

Maternal employment has a potential of improving household income. Most studies combined both formal and informal employment and discounted informal employment in the analysis^(27,28)^. Maternal informal employment (buying and selling, commercial farming and income generating projects) was protective of childhood stunting in this study. In contrast, an analysis of Uganda DHS data found children of mothers who were involved in agriculture to have increased odds of being stunted^(26)^. However, our study found unemployment in the father to increase the risk of childhood stunting. Not going to school (both mother and father) was seen as a significant predictor for childhood stunting in our study. A study conducted in India argues that maternal education improves the ability of mothers to understand and comprehend health messages^(29)^.

A double orphan had a higher likelihood of being stunted. While a majority of studies found no link between childhood stunting and child orphan status, very few classified child stunting as double or single orphan. A majority of studies found no association between childhood stunting and orphan status^(30,31)^. While the household size was not significantly associated with stunting in our study, we found having siblings regardless of number and an age difference of less than 24 months apart as highly likely to increase risk of childhood stunting. This association can similarly be explained by competing for attention from the mother hence diminished care for either sibling. An analysis of the demographic of health surveys in sub-Saharan Africa found the odds of stunting in children to increase as household size increased^(32)^. This study postulated that family size was linked to food availability and thus inversely affected the nutrition outcome of the growing child.

Children who were said to have never breastfed had a higher likelihood of being stunted. Breast milk is regarded as a preventive measure to childhood malnutrition^(33)^. However, the role of early initiation of breast milk has contrasting findings. A majority of studies find early initiation of breast milk as a significant protector to stunting^(32,34)^. Due to the chronic nature of stunting, the finding lacks biological plausibility. However, the relationship can be explained as a proxy for improved maternal attention^(34,35)^. Our study found no significant relationship between early initiation and childhood stunting.

Loss of appetite in children could be a sign of an underlying infection. Children who in the past 1 month have been having trouble with loss of appetite were more likely to be stunted. Similarly, children with diarrhoeal infection in the past month had higher rate of being stunted. Multiple studies from several geographical areas have found existence of a significant relationship between diarrhoea and childhood stunting^(36,37)^. Diarrhoea results in loss of fluids, affects the absorption of food in the stomach and can also cause loss of appetite^(38)^. Worm infection was also found to be significantly associated with child stunting. Worms feed on the food that was meant for the host, thereby depriving the host of sufficient nutrition. Additionally, they may also cause difficulties in absorption of food in the intestines as well as excrete harmful substances, all of which can result in increased odds of childhood stunting^(36)^. Access and utilisation of health services assessed through vaccination status, vitamin A status, ANC uptake and place of delivery showed that children with exposure to health facilities were less likely to be stunted. Caregivers who visit health facilities are more likely to receive health education which in turn is more likely to improve care giving practices and reinforce positive behaviours.

Water and sanitation remain one of the most important areas of focus in addressing child stunting. Numerous studies have assessed the link between water and sanitation to childhood stunting^(13,36)^. We found children who had unsafe water sources and who lived in households with no waste disposal pit to be more likely to be stunted. It has been argued that the association between water and sanitation and stunting is due to these factors being linked to other determinants of stunting such as household wealth and parental education^(39)^. However, there is a paucity of studies that have assessed if these factors remained significantly associated after controlling for wealth and education. The F-diagram often used in Participatory Health and Hygiene Education interventions explains the association between childhood stunting and water, sanitation and hygiene as highly linked to exposure to pathogens via the faecal–oral transmission route more often exacerbated by limited access to water and sanitation facilities^(40,41)^.

There are limited studies that analysed the role of support systems in the reduction of stunting in sub-Saharan Africa. In a study that looked at the role of grandmothers in sub-Saharan Africa, children who lived with grandparents were less likely to be stunted^(42)^. This association is similar to our finding that support from the maternal grandmothers was more likely to be protective of stunting in comparison to paternal grandmothers. A literature review on the role of grandparents globally highlighted similar results that show the grandparents’ role in reducing adverse growth outcomes including stunting^(43)^. A study conducted in Uganda that looked at voluntary community health clubs postulated that social learning and financial intelligence gained during interactions with other club members were more likely to assist mothers in caring for their child^(44)^. This is in agreement with our study where children with stunting were more likely to have mothers who were not part of a social club.

### Strengths and limitations

A case–control study design allows for an analysis of multiple factors at the same time and is cheaper in comparison with a longitudinal study. While a longitudinal study with a large sample size would be a better option to use in analysing factors associated with childhood stunting, we carried out a case–control study due to financial, time and human resource limitations. The study was not matched by possible confounders such as age, sex, household income among others so as to allow for analysis of whether the factors were significant predictors of stunting in Zimbabwe as there is limited information on their contribution in Zimbabwe. While the VHW registers are expected to record anthropometric information of all children within their village, some villages did not have a VHW. Additionally, the study did not assess completeness of the VHW register and there is likelihood the register may miss some children from the village. As such, the use of an incomplete sampling frame can introduce selection bias.

Some factors outlined in the UNICEF stunting framework were not assessed as they have been extensively studied in Zimbabwe and so as to reduce the time taken interviewing participants. Residual confounding from missing covariates such as household food security, genetic factors, political climate and parental anthropometric outcomes could be resolved through a longitudinal study. Our assessment of household wealth was calculated using the World Bank indices that were not tested for construct validity in Zimbabwe; further research is required to assess its reliability in assessing household wealth in this context. The four-star diet defined as a meal containing animal source foods, starch, legumes and vitamins was also not validated. The study did not take into consideration variation in children’s diet based on their age. Future studies may do well to assess the differences in dietary intake based on the child’s age while measuring its contribution to stunting. Purposive selection of districts and provinces done so as to represent the most and least affected population by childhood stunting reduces the generalisability of study findings to the Zimbabwean population. As the study is the first to consider a vast majority of factors outlined in the UNICEF stunting framework in Zimbabwe, it was assumed stepwise analysis would be the best model to use. Future studies may do well to consider using a multi-level regression analysis considering the fact that individuals are often clustered within households.

Recall bias may affect multiple questions in the present study due to its retrospective nature. Mothers may fail to recall whether they initiated breast-feeding for their child within 1 h or not. Questions on dietary patterns were limited to the previous 7 d to avoid recall bias. However, this may not accurately represent the relationship between diet and stunting as stunting is a chronic condition. A number of caregivers were not the child’s biological mother; hence, data on maternal factors such as maternal height and maternal depression were not adequately represented. Hence, future studies may do well to analyse the UNICEF stunting framework through a longitudinal analysis.

## Conclusion

This study has highlighted the role of the child’s gender and family factors such as parental age, and child spacing as strong predictors of stunting in children. There were numerous household-, social- and community-level factors that were also found to be significantly associated with childhood stunting. This study adds to the growing body of evidence supporting the UNICEF stunting framework and strengthens a need to focus multi-sectoral interventions with a multifactorial approach to effectively address stunting in high prevalence countries. Future studies may consider a cohort approach to understanding the stunting framework in a low-income setting such as Zimbabwe.
